# Operationalizing regional One Health initiatives in Southeast Asia: Ways forward

**DOI:** 10.1016/j.onehlt.2025.101034

**Published:** 2025-04-14

**Authors:** Steven Lâm, Sinh Dang-Xuan, Fred Unger, Tongkorn Meeyam, Phuc Pham-Duc, Supaporn Wacharapluesadee, Hung Nguyen-Viet

**Affiliations:** a International Livestock Research Institute, Nairobi, Kenya; b International Livestock Research Institute, Hanoi, Viet Nam; c Southeast Asia One Health University Network, Chiang Mai, Thailand; d Hanoi University of Public Health, Hanoi, Viet Nam; e Institute of Environmental Health and Sustainable Development, Hanoi, Viet Nam; f Thai Red Cross Emerging Infectious Diseases Clinical Center, King Chulalongkorn Memorial Hospital, Bangkok, Thailand

**Keywords:** One health, Operationalization, Regional workshop, Southeast Asia, Multisectoral coordination

## Abstract

Operationalizing One Health initiatives that link human, animal, and environmental health at the regional level is key for jointly addressing infectious diseases that can cross borders. This work is urgently needed in Southeast Asia, a recognized hotspot for emerging animal and human infectious diseases that have the potential to spread globally. As such, our objective is to identify action items to advance regional One Health efforts in Southeast Asia. We organized a 1.5-day workshop that convened 34 experts from government, national research institutes, universities, and international organizations spanning seven countries in Southeast Asia. Group discussions and prioritization exercises were conducted which led to 12 action items, serving as ideas for resourcing, operationalizing, and implementing One Health efforts in Southeast Asia. Participants also emphasized the importance of sustained funding, a collective voice, and a willingness among members to be bold in their collective efforts. Given the heightened focus on zoonotic risks in Southeast Asia, harnessing this momentum by operationalizing regional efforts could establish a solid foundation to draw on when facing future global health threats.

## 1 Introduction

Southeast Asia (SEA) is a hotspot for emerging zoonotic diseases due to a combination of factors such as intensive livestock production systems, high diversity of wildlife, rapid changes in land use, and impacts of climate change [1,2]. These dynamics foster high rates of contact among humans and animals – including livestock and wildlife – amplifying the risk of disease spillover. With the pandemic-potential outbreak of avian influenza in the 2000s and the more recent occurrence of COVID-19 declared in 2020, these instances illustrate that new, highly infectious pathogens periodically arise at the human-animal interface, a trend likely to persist [3].

Embracing a One Health approach offers a promising framework for tackling health threats stemming from the interface between humans, animals, and the environment. Acknowledging the interconnectedness of these elements, One Health calls for collaboration across sectors and disciplines [4]. Effective communication, coordination, and capacity building are key in this endeavour. However, the traditional separation of sectors and disciplines, coupled with inconsistent investments in One Health action, creates challenges for implementation [5,6].

Regional One Health initiatives have the potential to overcome siloed actions and resource constraints through joint activities and information sharing [7]. While such mechanisms to support their development have been established in certain regions (e.g. in West Africa, Latin America and the Caribbean) [8,9], regional commitments to establishing one are nascent in SEA [10]. To facilitate successful operationalization of regional One Health initiatives, experts in SEA have suggested that, among other factors, there exists a willingness to collaborate, which is already in place [11]. Of note, we refer to operationalization as establishing a structure or process, while implementation involves carrying out those processes.

SEA has a timely opportunity to collectively mitigate future disease outbreaks. This opportunity arises not only from a shared interest to work together but also from the region's common One Health priorities, which frequently extend beyond national borders. These priorities include zoonotic influenza, rabies, and food safety (Table 1). Additionally, there exists a legacy of One Health action, particularly evident in the early 2000s in the wake of Severe Acute Respiratory Syndrome (SARS) and highly pathogenic avian influenza (HPAI) epidemics [12]. Over the past five years (2019–2024) there have been at least 20 research, development, and research-for-development initiatives across SEA countries, focusing on similar topics (Table 2). Facilitating opportunities for experience sharing could help to strengthen these initiatives.

**Table 1:** National-level priority zoonotic diseases of countries in Southeast Asia.^a^

	Brunei	Cambodia	Indonesia	Laos	Malaysia	Myanmar	Philippines	Singapore	Thailand	Timor Leste	Vietnam
Zoonotic influenza	x	x	x	x	x	x		x	x	x	x
Rabies	x	x	x	x	x	x	x		x	x	x
Food safety^b^	x	x	x	x	x		x	x			x
Anthrax	x	x	x	x		x	x			x	x
Brucellosis		x			x			x		x	
Tuberculosis						x		x		x	
Ebola							x		x		
Corona viral diseases			x						x		
Japanese encephalitis						x	x				
Q fever					x						
Nipah virus									x		

a Data are from International Health Regulations (IHR) Joint External Evaluations conducted between 2017 and 2022 [13]. The IHR provides an overarching legal framework that defines countries' rights and obligations in handling public health events and emergencies that have the potential to cross borders. The identification of national zoonotic disease priorities facilitates the implementation of the IHR.

b Food safety issues include leptospirosis, trichinellosis, *Salmonella*, *Escherichia coli,* and *Streptococcus suis*.

**Table 2:** Overview of One Health initiatives in Southeast Asia that started from 2019 to 2024 (bold indicates countries in Southeast Asia).^a^

Timeline	Initiative	Type	Country	Objective	Funder, amount
**Zoonoses**					
2024–**2029**	Strengthening Local Health Security Activity	Development	**Vietnam**	To strengthen Vietnam's capacity at the subnational levels to effectively prevent, detect, and respond to emerging infectious diseases through One Health approaches.	United States Agency for International Development, N/A
2022–**2026**	Safety across Asia for the global environment	Research	**Thailand, Vietnam, Laos, Malaysia**	To prevent pandemics by focusing on the connection between wildlife trafficking and zoonotic disease transmission.	European Union, N/A
2023–**2026**	Preventing zoonotic disease emergence in Africa and Cambodia	Research	**Cambodia**, Cameroon, Guinea, Madagascar Senegal	To reduce this risk as much as possible to prevent the emergence of new epidemics.	French Development Agency, N/A
2020–**2025**	Emerging Infectious Diseases: Southeast Asia Research Collaboration Hub	Research	**Thailand,** **Malaysia,** **Singapore,** United States	To analyze the diversity of key viral pathogens in wildlife, the frequency and causes of their spillover.	United States National Institute of Allergy and Infectious Diseases, N/A
2020–**2025**	Strategies to Prevent Spillover	Research-for-development^b^	Bangladesh, **Cambodia,** Liberia, Sierra Leone, Uganda, **Vietnam**	To strengthen the capacities of priority countries to identify, assess, and monitor risks associated with emerging zoonotic viruses and to develop risk reduction measures.	United States Agency for International Development, USD 100 million
2021–**2025**	Livestock enhancement through ecohealth/One Health assessment in South-East Asia	Research	**Philippines, Indonesia, Laos**	To identify the socio-economic drivers that link the animal-human-animal interfaces at the local and national levels for the effective operationalization of a unified One Health action in Southeast Asia.	Australian Centre for International Agricultural Research/Canada's International Development Research Centre, AUD 997,838
2021–**2025**	Policy support to the Philippines' National Surveillance and Control Programs	Research	**Philippines**	To provide policy support to the Philippines' National Surveillance and Control Programs.	Australian Centre for International Agricultural Research/Canada's International Development Research Centre, AUD 1,000,000
2021–**2025**	Developing strategies to reduce brucellosis transmission based on One Health collaboration	Research	**Timor-Leste**	To identify risk factors for bovine brucellosis transmission and facilitate implementation of evidence-based brucellosis control measures in Timor-Leste using a participatory and One Health approach.	Australian Centre for International Agricultural Research/Canada's International Development Research Centre, AUD 999,943
2022–**2025**	Cambodia Pandemic Prevention, Preparedness and Response Project	Development	**Cambodia**	To increase preparedness, prevention, and response to health emergencies with a pandemic potential in Cambodia through a One Health approach.	Pandemic Fund, USD 19.5 million
2022–**2025**	Improving human health through sustainable value chains using ICT in Vietnam	Research-for-development	**Vietnam**	To apply a One Health approach to ensure a multidisciplinary and multi-sectoral approach to addressing the threat of zoonotic diseases.	Korean Ministry of Agriculture, Food and Rural Affairs, USD 3 million
2021–**2024**	Global Programme for Pandemic Prevention and Response, One Health	Development	**Cambodia**	To eliminate deaths from rabies by 2030 in alignment with the global strategy “Zero by 30”.	German Federal Ministry for Economic Cooperation and Development, N/A
2022–**2024**	CGIAR One Health Initiative^c^	Research-for-development	**Vietnam**	To address the interspecies transmission risks in wildlife value chains and to develop food safety intervention packages in wet markets.	CGIAR Trust Fund, USD 1.1 million
2023–**2024**	Immediate technical assistance for the Ministry of Agriculture for Animal and One Health systems	Development	**Indonesia**	To support prevention, preparedness, and response to zoonotic diseases to pre-empt the emergence and mitigate the impact of these threats.	United States Agency for International Development, USD 380,000
2023–**2024**	Global Health Security Project	Development	**Indonesia**	To minimize the risk and impact of emerging diseases and pandemics by pre-empting outbreaks and enhancing ability in prevention, detection, and response.	United States Agency for International Development, USD 2.5 million

**Food security**					
2023–**2027**	ASEAN-CGIAR Innovate for Food Regional Program	Research-for-development	**Cambodia, Philippines, Laos, Vietnam, Malaysia**	To scale up and out bold integrated innovations that will enhance the resilience of ASEAN's agri-food systems to climate change.	Australian Centre for International Agricultural Research/United Kingdom government, N/A
2024–**2027**	Sustainable Wildlife Management Asia	Research-for-development	**Vietnam, Laos, Indonesia**	To improve wildlife conservation and food security.	European Union, USD 2 million
2020–**2025**	Agroecology and Safe Food System Transitions in Southeast Asia	Research-for-development	**Cambodia, Laos, Myanmar, Vietnam**	To make food and agricultural systems in Southeast Asia more sustainable, safer, and inclusive through harnessing the potential of agroecology.	French Development Agency/European Union/French Facility for Global Environment, N/A

**Antimicrobial resistance**					
2022–**2027**	Transformational Strategies for Farm Output Risk Mitigation	Research-for-development	**Vietnam, Indonesia**	To harness private sector-led innovation to address emerging infectious diseases and antimicrobial resistance in animal value chains.	United States Agency for International Development, N/A
2019–**2024**	Vietnam Fleming Fund Country Grant	Development	**Vietnam**	To identify and address gaps in the surveillance of antibiotic-resistant bacteria in Vietnam.	Fleming Fund Country Grant, £ 8,948,514

**Environment**					
2021–**2025**	The role of agricultural and forest landscapes on human and environmental health in Cambodia	Research	**Cambodia**	To understand the importance of forest plants in the diet and health of Cambodian people and how exposure to agricultural chemicals might threaten this.	Australian Centre for International Agricultural Research/Canada's International Development Research Centre, AUD 999,999

a This overview is not meant to be comprehensive but illustrative of examples of existing One Health initiatives; it was developed based on a rapid scan of the literature as well as knowledge from the authorship team, all of whom were a part of an initiative.

b Research-for-development is conceptualized as conducting research that is not only scientifically rigorous but also directly contributes to development outcomes, such as improving the livelihoods of smallholder farmers [14].

c We placed the CGIAR One Health Initiative under zoonoses but acknowledge it also addresses the themes of food security and antimicrobial resistance [15].

With the increased attention to zoonotic risks in Southeast Asia and the shared interest in regional, multisectoral action to address these risks, this piece aims to identify strategies to finance, operationalize, and implement regional One Health efforts. To set the context, we first scan the literature on One Health action in SEA (for the review approach, see Supplementary File Table S1). We then present the results of a regional workshop to share experiences from implementation, and building on this exchange, identify action items to translate commitments to working together regionally into tangible outcomes. We hope this piece can provide a resource not only for SEA but also for other regions looking to address complex health challenges that transcend national borders and require multisectoral solutions.

### 1.1 One Health actions in Southeast Asia

From the literature review, there is growing acknowledgment among countries in SEA of the importance of building One Health capacity nationally. Regional initiatives, such as the Tripartite mechanism involving the Food and Agriculture Organization (FAO), World Organization for Animal Health (WOAH), and World Health Organization (WHO), have been established to promote such capacity building. This mechanism has been operational in the Asia-Pacific region since 2011 [6]. Evolving into the Quadripartite, it expanded its scope by signing a Memorandum of Understanding with the United Nations Environment Programme (UNEP) in 2022. A substantial contribution has been the organization of eight Asia-Pacific workshops on multisectoral collaboration for the prevention and control of zoonoses since 2010. Furthermore, in 2023, the Quadripartite supported Laos, Cambodia, and Vietnam in conducting International Health Regulation – Performance Veterinary Services National Bridging Workshops, bringing together national stakeholders from animal and human health services to strengthen collaborations [16].

**Fig. 1:**
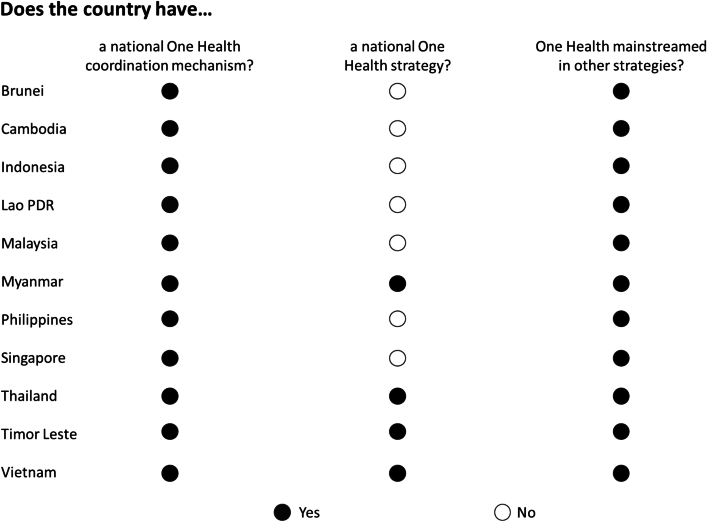
Stocktake of One Health coordination mechanisms and strategies in Southeast Asia.

All countries in SEA today have One Health mechanisms at the national level, serving as a fundamental basis for regional coordination (Fig. 1). Mechanisms exist at varying levels of formality, from informal (e.g. Timor Leste) to institutionalized (e.g. Cambodia). Notably, only a few countries have action plans outlining priorities and strategies for implementation (e.g. Myanmar, Vietnam, Thailand); however, all countries have “mainstreamed” One Health by developing plans on antimicrobial resistance (AMR) and/or infectious diseases that call for the use of a One Health approach (for details see Supplementary File Table S2).

One Health coordination is increasingly promoted at the regional level, supported by regional organizations including the Association of Southeast Asian Nations (ASEAN), Asia-Pacific Economic Cooperation, and the Southeast Asia One Health University Network (SEAOHUN). For instance, operating as a regional university network, SEAOHUN has been facilitating the sharing, connection, and cooperation in One Health education for current and future One Health workforces in SEA since 2011. Funders and international and national development agencies have also played a role in promoting regional action by developing region-focused One Health guidance [17] and frameworks [[18], [19], [20]]. A notable example is the 2023 Asia Pacific Health Security Action Framework – built upon its predecessor, the 2005 Asia Pacific Strategy for Emerging Diseases – which provides a common framework for governments, donors, and partners to work collectively towards regional health security by ensuring functional national and regional systems are in place for preparedness prevention and response [18].

There are also growing commitments to One Health coordination at a global level, spearheaded by SEA countries. For example, the Global Health Security Agenda was launched in Feb 2014 to advance a world safe from infectious disease threats [21]; in the Asia-Pacific region, there are leading countries for three action packages: zoonotic diseases (led by Indonesia and Vietnam); national laboratory systems (led by Thailand); and, workforce development (led by Thailand). As an overarching approach, these action packages emphasize the importance of cross-border collaborations to ensure key information is shared quickly in times of outbreaks.

## 2 Methods

### 2.1 Regional workshop on One Health financing, operationalization, and implementation in Southeast Asia

The International Livestock Research Institute (ILRI) and SEAOHUN co-organized a 1.5-day workshop in Bangkok, Thailand, from December 19 to 20, 2023. ILRI is a CGIAR centre dedicated to advancing food security and reducing poverty through research on the efficient, safe, and sustainable use of livestock, while SEAOHUN is a collaborative network of regional universities working to enhance the capacity of the One Health workforce.

The workshop aimed to exchange experiences and generate ideas for regional One Health investment, operationalization, and implementation. Bringing together 34 participants from Cambodia, Indonesia, Laos, Malaysia, the Philippines, Thailand, and Vietnam, the diverse group included researchers, representatives from the government (such as those from the Department of Livestock Development, Department of Disease Control), representatives from international organizations (such as those from WOAH, WHO, UNEP), and donors such as World Bank. Participants held expertise in various domains, including public health and animal, plant, and environmental sciences. Most came from biological science backgrounds, with limited representation from the social sciences.

The workshop began with presentations by participants on One Health initiatives nationally and regionally. Experiences were then synthesized and presented narratively. Afterward, several group discussions were held to identify strategies for resource mobilization, operationalization, and implementation, all grounded in the participants' own experiences. The final session included a prioritization exercise to agree on a set of priority action items. It applied an adapted nominal group technique involving structured group discussions [22]. Specifically, participants were split into four groups and asked to individually contribute ideas to generate a list; then, participants discussed and added new ideas as appropriate. Afterward, participants prioritized ideas with justifications. Finally, groups came back together to present the key ideas, which were compiled into a new list.

### 2.2 Ethics approval and consent to participate

This study obtained ethical approval from the Institutional Review Board of the Hanoi University of Public Health (No. 458/2022/YTCC-HD3). All research was performed in accordance with this institution's guidelines. All workshop participants provided informed consent after being briefed on the nature of the workshop, the use of their data, and their rights.

## 3 Results

### 3.1 Experiences of One Health implementation in Southeast Asia

Workshop attendees highlighted their ongoing engagement in several regional and global platforms dedicated to sharing knowledge on diverse One Health topics, primarily zoonoses (Table 3). Participants also shared key networks for learning about One Health capacity-building opportunities, including SEAOHUN, the Asia Pacific Consortium of Veterinary Epidemiology network, and One Health Commission. While these platforms reportedly provided valuable opportunities to exchange knowledge and events, participants stressed the need for formalized structures that could lead to joint action that goes beyond information sharing.

**Table 3:** One Health platforms in Southeast Asia.

Platform	Description	Established by: organization (year)	Current status or outcomes
**Zoonoses**			
Preventing Zoonotic Diseases Emergence (PREZODE)	An international initiative promoting prevention, early detection, and resilience to rapidly respond to emerging infectious diseases of animal origin.	French Development Agency (2021)	As of January 2025, PREZODE encompassed more than 260 organizations [24].
Gestion des Risques Emergents en Asie du Sud-Est (GREASE) network	A regional network that supports research activities for better management of emerging epidemic risks in Southeast Asia.	French Agricultural Research Centre for International Development (2009)	GREASE has provided scientific and institutional support to facilitate interactions between various stakeholders [25].
Mekong Basin Disease Surveillance (MBDS)	A self-organized, sub-regional network to strengthen national and sub-regional capabilities in infectious disease surveillance and outbreak response.	Governments in Cambodia, China, Laos, Myanmar, Thailand, and Vietnam (2021)	MBDS has contributed to cross-border communications, epidemiological capacity building, and outbreak containment [26].
Asian Partnership on Emerging Infectious Disease Research (APEIR)	A research network to support activities on knowledge generation, research capacity building, and policy and social advocacy.	Canada International Development Research Centre and governments and researchers in Cambodia, Laos, Indonesia, Thailand, Vietnam and China (2006)	APEIR has completed regional research projects that have yielded a number of outputs to inform disease response [12].
Global Outbreak Alert and Response Network (GOARN)	A global technical and operational partnership was established as a key mechanism to engage the resources of technical agencies for rapid identification, confirmation, risk assessment, and response to public health emergencies of international importance.	World Health Organization (2000)	As of February 2024, GOARN has more than 300 partners [27].

**Food security**			
International Food Safety Authorities Network (INFOSAN)	A global network of 186 national food safety authorities that assists Member States in managing food safety risks.	Food and Agriculture Organization, World Health Organization (2004)	INFOSAN has facilitated the rapid exchange of information across borders during hundreds of food safety events [28].
Market and Agriculture Linkages for Cities in Asia platform (MALICA)	A collaborative platform aims to strengthen the research and decision capacity on food market analysis and urban/rural linkages of researchers, students, public officials, and private groups in Vietnam and Laos.	French Agricultural Research Centre for International Development together with agricultural research institutes in Vietnam (2002)	MALICA advanced activities in governance, research, training, and policy dialogue [29].

**Antimicrobial resistance**			
Global Antimicrobial Resistance and Use Surveillance System (GLASS)	A global collaborative effort to standardize antimicrobial resistance surveillance.	World Health Organization (2015)	From 2017 to 2020, GLASS facilitated a 15 % increase in countries reporting antimicrobial resistance data [30].
Joint Programming Initiative on Antimicrobial Resistance (JPIAMR)	A global collaborative organization and platform, engaging 29 nations to curb antimicrobial resistance with a One Health approach.	European Union (2013)	JPIAMR coordinated national research funding [31].

**Wildlife**			
International Alliance Against Health Risks and Wildlife Trade	An inclusive and interdisciplinary platform aiming to reduce health risks from wildlife trade and consumption through a One Health approach.	German Corporation for International Cooperation (2021)	As of February 2024, the community consisted of more than 120 organizations [32].
Regional Wildlife Health Network for Asia and the Pacific	A network to strengthen One Health, multi-sectoral collaboration, and capacity for wildlife health management, monitoring, and surveillance systems.	World Organization for Animal Health (2021)	Sub-regional wildlife health networks met regularly to discuss key issues [33].

Notably, there already exists structures for cross-border cooperation, which provide a foundation to develop mechanisms for regional One Health coordination. For example, ASEAN member countries established the ASEAN Centre for Biodiversity (ACB) in 2005, the ASEAN Centre for Public Health Emergencies and Emerging Diseases (ACPHEED) in 2020, and the ASEAN Centre for Animal Health and Zoonoses (ACCAHZ) in 2021, highlighting commitments to address health concerns that require multisectoral coordination. Further, ASEAN committed to establishing a One Health Network in 2023 (ASEAN 2023). However, timelines for agreements to take effect can be lengthy, as seen in the five years it took for the ACPHEED agreement to come into force [23].

SEA reportedly does not receive sustained funding for its One Health activities which means that initiatives tend to be one-off rather than long-term. While governmental departments contribute some funds (e.g. the Ministry of Public Health in Thailand), primary financial backing comes from external donors. These donors include international development agencies (e.g. from the United States, France, Canada, Australia, the United Kingdom, and Germany), global funds (e.g. Pandemic Fund to Cambodia), platforms (e.g. GREASE network), and private companies (e.g. Cargill in Vietnam). Participants stressed that ideally, member countries would prioritize national ownership and financial sustainability of a regional One Health initiative while partner organizations would offer technical support.

Apart from the challenge of sustaining funding, participants from different countries highlighted several other hurdles that exist at the national level. These include limited infrastructure to share data (Laos), insufficient capacity (Cambodia), buy-in (Malaysia), and bureaucratic hurdles (Vietnam). These factors create difficulties in cross-country coordination. Participants highlighted several strategies for overcoming these challenges – including applying community-based approaches (Laos, Vietnam), building capacity (Philippines, Cambodia), and learning from regional/global initiatives (Vietnam, Thailand). Participants also emphasized that there is a conducive environment for a regional body to support the implementation of these strategies.

### 3.2 Proposed action items to propel regional One Health action in Southeast Asia

Participants suggested action items for financing, operationalizing, and implementing regional One Health initiatives, which were summarized in Box 1 and elaborated on below:

1) **Leverage existing and emerging funding sources.** Draw support not only from existing donors but also from emerging sources such as the Global Fund, the Fleming Fund, the Asian Development Bank, the Bill and Melinda Gates Foundation, international development agencies (e.g. from Denmark and Japan), as well as various private companies with interests in community health (e.g. Chevron-SEAOHUN collaboration) and animal health (e.g. Dabaco in Vietnam). In addition, aligning One Health with global sustainable development targets and agendas such as climate action presents opportunities for securing additional funding.
2) **Encourage domestic resource mobilization.** While donors currently play a key role in financing One Health activities in SEA, balancing global health priorities and national financing is crucial for sustainability. Participants highlighted the promise of co-funding, such as Vietnam organizing rabies awareness-building activities in September 2023, with a national financial contribution of 30 %, complemented by 70 % from donors. Although the in-kind contributions from the countries are appreciated, participants suggested countries prioritize One Health more and allocate budgets for One Health action.
3) **Cultivate private-public partnerships.** Develop a strategy to engage the private sector to secure funding, drawing insights from successful engagement models, and highlighting the advantages of embracing a One Health approach within their core activities. The private sector can contribute not only financial resources but also expertise and networks.
4) **Develop business cases for One Health.** There is an ongoing need for empirical evidence regarding the social and economic impacts of One Health action, as well as the cost-effectiveness of interventions. At the same time, there is a continued need to formulate business cases for One Health investments drawing on these studies.
5) **Establish a One Health centre for Southeast Asia.** It is essential to have a mechanism to facilitate the development of regional initiatives. Drawing inspiration from the One Health Centre in Africa [34], which collaborates closely with the African Union to support eight countries, this model could be considered for SEA. Alternatively, rather than creating a new structure, SEAOHUN could serve as a regional One Health centre, but further groundwork is required, including clarifying comparative advantages as well as expanding its scope to consider not only capacity building but also research and outreach initiatives. ASEAN may also consider integrating One Health within existing centres (e.g. ACPHEED, ACCAHZ) as well as accelerate the establishment of the ASEAN One Health Network. Existing multilateral frameworks, such as the Quadripartite One Health Joint Plan of Action, could support this process by providing technical support.
6) **Develop data governance frameworks.** Establishing data governance frameworks for use among One Health sectors within the SEA region could encourage data and information sharing by guiding the structure and function of a mechanism to support regional One Health initiatives. Ideas could come from existing frameworks already developed in other regions (e.g. West Africa, Latin America and the Caribbean) [8,9].
7) **Formalize national coordination mechanisms.** In several countries, One Health coordination is currently informal. It is important for mechanisms to be institutionalized via an enabling policy – involving establishing a clear mandate and allocating resources – thereby improving the sustainability of mechanisms. These structures could be key resources for regional efforts to draw on. As such, regional efforts could help to build this resource by facilitating the sharing of institutionalization experiences.
8) **Integrate social sciences.** Regional efforts should broaden their scope beyond immediate zoonoses disease prevention and control, taking into account factors such as social determinants of health, cultural practices, and human behavior. For instance, poverty underlies the vulnerability of marginalized communities to One Health issues, impacting specific populations more than others, and this warrants attention. Social scientists specialize in understanding human behaviours and dynamics and can thus contribute to the development of inclusive engagement strategies.
9) **Strengthen engagement with diverse dimensions of One Health.** It is essential to recognize that conversations on One Health extend beyond the confines of the human or animal health sector; sectors such as plant health, wildlife, conservation, and environment are actively participating in similar discussions. Engaging broader sectors helps to prevent the risk of addressing issues in isolation, potentially leading to their resurgence elsewhere. Ultimately, this approach saves time, resources, and lives.
10) **Integrate One Health into national and local frameworks.** Adopting One Health measures not only aligns with broader social and economic objectives related to well-being and livelihoods but also strengthens national plans, such as those focused on food systems, rural development, and climate action. Furthermore, for regional efforts to be effective, the impact must ultimately extend to communities, and a community-based approach is integral to this process. The establishment of One Health field sites in some countries has been helpful; in Thai Nguyen province in Vietnam, students supported local communities through field sites focused on addressing AMR and food safety. This effort initially unfolded in one district and has now expanded to cover additional districts.
11) **Take stock of past and current initiatives.** Establishing a repository of One Health initiatives – including data on best practices – could help to facilitate the sharing of experiences. Importantly, this would require initiatives to document these experiences. One approach to obtaining this data involves conducting process evaluations to uncover insights for enhancing implementation, alongside outcome evaluations to gauge the extent to which initiative objectives were achieved. Moreover, in maintaining a repository, regular analysis of its contents could aid in identifying promising research avenues and methodologies, stimulating academic and practical inquiry.
12) **Invest in the One Health workforce.** As the current and next generations of One Health professionals are key to managing future outbreaks effectively, there is a need to continue investing in the workforce to ensure the availability of necessary competencies. Advocating for funding and support to training programs already being implemented by SEAOHUN and other regional entities can help to ensure their sustainability.

Box 1 **Action items for financing, operationalizing, and implementing One Health initiatives in Southeast Asia.**

**Table:** 

**Resource mobilization** 1) Leverage existing and emerging funding sources. 2) Encourage domestic resource mobilization. 3) Cultivate private-public partnerships. 4) Develop business cases for One Health. **Operationalization** 5) Establish a One Health centre for Southeast Asia. 6) Develop data governance frameworks. **Implementation** 7) Formalize national coordination mechanisms. 8) Integrate social sciences. 9) Strengthen engagement with diverse dimensions of One Health. 10) Integrate One Health into national and local frameworks. 11) Take stock of past and current initiatives. 12) Invest in the One Health workforce.

Alt-text: Box 1

## 4 Discussion

During a 2023 regional workshop, representatives from various sectors and disciplines across Southeast Asia proposed 12 strategies for financing, operationalizing, and implementing regional One Health efforts. Particularly emphasized in financing, and spanning all areas, was the importance of sustained funding. Ideally, governments in SEA would prioritize national ownership and financial sustainability of a regional One Health effort, while international partner organizations would offer technical support, aligning with models shown to be effective from other regions like West Africa and Latin America and the Caribbean [8,9]. Furthermore, the existence of similar regional coordination mechanisms elsewhere suggests that establishing one in SEA is both feasible and timely.

Participants also emphasized the need for a formal regional presence to work effectively. This sentiment resonates with perspectives expressed in other studies concerning how coordination of zoonotic disease control – the One Health issue currently receiving the most attention – could be best operationalized in SEA [11,35,36]. We build on these studies by suggesting potential entry points, such as creating a new One Health centre for SEA or integrating regional One Health efforts into an existing body's portfolio (e.g. SEAOHUN, ASEAN). Furthermore, the One Health Joint Plan of Action (2022–2026) developed by the Quadripartite provides a useful framework not only for establishing One Health efforts nationally but also regionally.

In terms of implementation, national coordination mechanisms are present in all countries, but further development is needed so that they can contribute effectively to regional efforts. In 2014, the regional response to AMR got a boost when it was identified as one of the flagship priority areas of WHO in the SEA region [37]. Since then, WHO has been guiding member states to improve implementation of AMR national action plans, which included adopting One Health approach as a key strategy. Regional efforts can draw lessons from this implementation example by identifying priority areas and advocating for them, as well as providing guidance and support in addressing these areas effectively.

While regional efforts can provide high-level support, participants stressed that national governments must take initiative as well. Participants shared obstacles in implementing One Health actions in their own country related to maintaining funding, sharing data, building capacity, gaining buy-in, and navigating bureaucracy, consistent with findings from previous literature documenting experiences in Vietnam, Thailand, and Myanmar [5,38,39]. In addition to echoing these experiences, this discussion underscored opportunities for regional efforts to help address national-level challenges by facilitating the exchange of best practices and fostering cross-border initiatives.

## 5 Conclusion

The outlined ways forward serve as strategies for resourcing, operationalizing, and implementing regional One Health efforts in SEA, with relevance to other regions that are tackling complex health challenges requiring multisectoral approaches. The consensus among the assembled experts during the workshop was that such collaborative efforts are attainable provided there is sustained funding, a formal collective voice, and a willingness among members to pursue bold agendas. The heightened attention to zoonoses risks which have the potential for global transmission creates a conducive environment for action by governments in SEA and beyond. By leveraging this momentum through the implementation of regional One Health initiatives, we lay a foundation to address future global health threats.

## CRediT authorship contribution statement

**Steven Lâm:** Writing – review & editing, Writing – original draft, Methodology, Formal analysis, Conceptualization. **Sinh Dang-Xuan:** Writing – review & editing. **Fred Unger:** Writing – review & editing. **Tongkorn Meeyam:** Writing – review & editing. **Phuc Pham-Duc:** Writing – review & editing. **Supaporn Wacharapluesadee:** Writing – review & editing. **Hung Nguyen-Viet:** Writing – review & editing, Conceptualization.

## Consent for publication

Not applicable.

## Ethics approval and consent to participate

This study obtained ethical approval from the Institutional Review Board of the Hanoi University of Public Health (No. 458/2022/YTCC-HD3). All research was performed in accordance with this institution's guidelines. All workshop participants provided informed consent after being briefed on the nature of the workshop, the use of their data, and their rights.

## Funding

The workshop was co-hosted by the Southeast Asia One Health University Network and the International Livestock Research Institute, and co-funded by the CGIAR Initiative on One Health and the ASEAN-CGIAR Innovate for Food and Nutrition Security Regional Program. CGIAR research is supported by contributions to the CGIAR Trust Fund.

## Data availability

Data sharing is not applicable to this article as no datasets were generated or analysed during the current study.

## Declaration of competing interest

The authors declare that they have no known competing financial interests or personal relationships that could have appeared to influence the work reported in this paper.

## Data Availability

Data will be made available on request.

## Appendix A Supplementary data

Supplementary material Image 1
