# Supplementary material for: Operationalizing regional One Health initiatives in Southeast Asia: Ways forward
Source: One Health. 2025 Apr 14;20:101034. doi: 10.1016/j.onehlt.2025.101034 (PMC12059403; doi:10.1016/j.onehlt.2025.101034)
Supplement: Supplementary file 1 — Supplementary material [file mmc1.docx]

**Supplementary file**

| **Box S1.** Review approach. |
| --- |
| We conducted a rapid review of the published literature to compile information on One Health mechanisms, strategies, and action plans in Southeast Asia, following a systematic process for literature searching and selection ^1^. Our search on Google search engine employed a combination of keywords, including interministerial, intersectoral, multisectoral, coordination, framework, strategy, plan, One Health, Southeast Asia, Brunei, Cambodia, Indonesia, Laos, Malaysia, Myanmar, Philippines, Singapore, Thailand, Timor Leste, and Vietnam. Using the same search strategy, we also searched institutional websites of key organizations working in One Health, including World Organization for Animal Health (WOAH), World Health Organization (WHO), United Nations Environment Program (UNEP), and Food and Agriculture Organization (FAO). No search restrictions were placed. Searches were performed on 5 December 2023. We captured general characteristics of the article/report, including authorship details, year of publication, and country, as well as details relating to the One Health mechanism, strategy, and plan. |

**Table S1.** Overview of One Health mechanisms, strategic plans/frameworks, and mainstreaming initiatives in Southeast Asia.

|  | One Health mechanism | One Health plan or framework | Integration of One Health in antimicrobial resistance (AMR) plans | Integration of One Health in disease plans |
| --- | --- | --- | --- | --- |
| Brunei | [National Committee for International Health Regulations Implementation](https://iris.who.int/bitstream/handle/10665/332937/9789240006973-eng.pdf?sequence=1) (established 2018) | N/A | One Health is integrated into national strategic plan on [AMR](https://www.who.int/publications/m/item/brunei-darussalam-antimicrobial-resistance-national-action-plan) (2019-2023) | N/A |
| Cambodia | [Inter Ministerial Coordination Committee for One Health](https://www.who.int/westernpacific/news-room/feature-stories/item/who-champions-a-one-health-approach-when-responding-to-outbreaks) (established 2023) | N/A | One Health is integrated into national strategic plan on [AMR](https://www.fao.org/faolex/results/details/en/c/LEX-FAOC199420/#:~:text=This%20Multi%E2%80%90Sectoral%20Action%20Plan,a%20country%20with%20a%20healthy) (2019-2023) | N/A |
| Indonesia | [One Health Coordination Working Group](https://agrilinks.org/post/indonesia-prepares-face-future-epidemics-institutionalizing-one-health-approach) (established in 2021) | N/A | One Health is integrated into national strategic plan on [AMR](https://www.who.int/publications/m/item/indonesia-second-national-action-plan-on-antimicrobial-resistance-2020-2024) (2017-2019; 2020-2024) | N/A |
| Laos | [One Health Interministerial platform](https://www.grease-network.org/content/download/5736/42866/version/1/file/2.4_Vannaphone_OH+platform+in+Laos.pdf) (established in 2018) | N/A | One Health is integrated into national strategic plan on [AMR](https://cdn.who.int/media/docs/default-source/antimicrobial-resistance/amr-spc-npm/nap-library/%E0%BB%81%E0%BA%9C%E0%BA%99%E0%BA%8D-%E0%BA%94%E0%BA%97%E0%BA%B0%E0%BA%AA%E0%BA%B2%E0%BA%94-4.11.2019-new.pdf?sfvrsn=35808032_1&download=true) (2019-2023) | One Health is integrated into national strategic plan on [emerging infectious diseases](https://extranet.who.int/sph/sites/default/files/document-library/document/JEE%20Report%20Lao%20People%27s%20Democratic%20Republic%202017.pdf) (2016-2020) |
| Malaysia | [Ministerial Committee on the Control of Zoonotic Diseases](https://extranet.who.int/sph/sites/default/files/document-library/document/JEE%20Report%20Malaysia%202019.pdf) (established in 1999) | N/A | One Health is integrated into national strategic plan on [AMR](https://www.who.int/publications/m/item/malaysia--malaysian-second-action-plan-on-antimicrobial-resistance-(myap-amr)) (2017-2021; 2022-2026) | One Health is integrated into national strategic plan on [emerging diseases](https://www.moh.gov.my/moh/resources/Penerbitan/Garis%20Panduan/Pengurusan%20KEsihatan%20&%20kawalan%20pykit/MySED_II.pdf) (2017-2021) |
| Myanmar | [National Multisectoral Coordination](https://rr-asia.woah.org/wp-content/uploads/2020/05/ihr-myanmer-roadmap.pdf)  [Committee for One Health](https://rr-asia.woah.org/wp-content/uploads/2020/05/ihr-myanmer-roadmap.pdf) (established 2020) | [National One Health Strategic Framework and Action Plan of Myanmar](https://www.myanmardigitalnewspaper.com/en/question-implementing-national-strategic-plan-one-health-concerning-relations-human-and-animal#:~:text=In%20the%20National%20One%20Health,prioritize%20and%20are%20being%20implemented) (2019-2023) | - One Health is integrated into national strategic plan on [AMR](https://www.fao.org/faolex/results/details/en/c/LEX-FAOC202553/) (2017-2022) | N/A |
| Philippines | [Philippine Inter-agency Committee on Zoonoses](https://www.da.gov.ph/wp-content/uploads/2020/11/doh_da_denr_s2020.pdf) (established 2011) | N/A | One Health is integrated into national strategic plan on [AMR](https://cdn.who.int/media/docs/default-source/antimicrobial-resistance/amr-spc-npm/nap-library/philippine-national-action-plan-on-amr-2019-2023-final.pdf?sfvrsn=8bbe1fdb_1) (2019-2023) | One Health is integrated into national strategic plan on [zoonoses](https://www.who.int/philippines/news/detail/20-09-2022-one-health-approach-in-the-philippines) (2023-2028) |
| Singapore | [One Health Coordinating Committee](https://www.moh.gov.sg/docs/librariesprovider5/joint-one-health-report/joint-oh-quarterly-report_nov-2022.pdf) (established 2012) | N/A | One Health is integrated in national strategic plan on [AMR](https://www.fao.org/faolex/results/details/en/c/LEX-FAOC171511/#:~:text=The%20key%20strategic%20areas%20requiring,include%20all%20animal%20production%20sectors%3B) (2017-present) | N/A |
| Thailand | [Coordinating Unit for One Health](https://thaionehealth.org/) (established 2014) | - Thailand One Health Action Plan (2023-2027) (recently approved) | One Health is integrated into national strategic plan on [AMR](https://rr-asia.woah.org/wp-content/uploads/2020/03/thailand_thailands-national-strategic-plan-on-amr-2017-2021.pdf) (2017-2021) | One Health is integrated into national strategic plan on [emerging infectious diseases](https://faolex.fao.org/docs/pdf/tha169875.pdf) (2012-2016; 2017-2021) |
| Timor Leste | [One Health Working Group](https://extranet.who.int/sph/sites/default/files/document-library/document/JEE%20Report%20Democratic%20Republic%20of%20Timor-Leste%202018.pdf) (established 2018) | - [National One Health strategic framework](https://extranet.who.int/sph/sites/default/files/document-library/document/JEE%20Report%20Democratic%20Republic%20of%20Timor-Leste%202018.pdf) (2018) | One Health is integrated into national strategic plan on [AMR](https://www.fao.org/faolex/results/details/en/c/LEX-FAOC169512/#:~:text=These%20strategic%20objectives%20are%3A%20Objective,incidence%20of%20infection%20through%20effective) (2017-2020) | N/A |
| Vietnam | [National One Health Partnership for Zoonoses](https://onehealth.org.vn/) (2016-2020; 2021-2015) | [Vietnam One Health Strategic Plan for zoonotic diseases](https://onehealth.org.vn/upload/upload/National%20One%20Health%20Strategic%20Plan%20for%20Zoonotic%20Diseases_EN.pdf) (2016-2020; 2021-2025) | One Health is integrated into national strategic plan on [AMR](https://lawnet.vn/en/vb/Decision-1121-QD-TTg-2023-The-national-strategy-for-prevention-and-control-of-drug-resistance-8DDD4.html?tab=3) (2013-2020; 2023-2030) | N/A |

**References**

1 Haby MM, Chapman E, Clark R, Barreto J, Reveiz L, Lavis JN. What are the best methodologies for rapid reviews of the research evidence for evidence-informed decision making in health policy and practice: A rapid review. Heal. Res. Policy Syst. 2016. DOI:10.1186/s12961-016-0155-7.
